# Case Report: A neonatal case of *Elizabethkingia anophelis* sepsis complicated by meningitis and intestinal necrosis: a triumph of multidisciplinary management

**DOI:** 10.3389/fped.2026.1920161

**Published:** 2026-09-23

**Authors:** Ziao Zhang, Dan Zhou, Xuwei Tao

**Affiliations:** 1 School of Medicine, Department of Medicine, Wuhan University of Science and Technology, Wuhan, China; 2 Division of Neonatology, Wuhan Children's Hospital (Wuhan Maternal and Child Healthcare Hospital), Tongji Medical College, Huazhong University of Science & Technology, Wuhan, Hubei, China; 3 Division of Outpatient, Wuhan Children's Hospital (Wuhan Maternal and Child Healthcare Hospital), Tongji Medical College, Huazhong University of Science & Technology, Wuhan, Hubei, China

**Keywords:** elizabethkingia anophelis, metagenomic next-generation sequencing, multidisciplinary collaboration, neonate, sepsis

## Abstract

This case reports a 10 day old neonate with fulminant *Elizabethkingia anophelis* sepsis presenting as disseminated infection involving meningitis and intestinal necrosis. Pathogen identification was achieved on hospital day 2 by metagenomic next generation sequencing and confirmed by blood and cerebrospinal fluid cultures, enabling antimicrobial susceptibility guided therapy. The infant was treated with cefoperazone sulbactam plus levofloxacin on day 3, switched to levofloxacin plus trimethoprim sulfamethoxazole (TMP SMX) on day 10, and stepped down to oral TMP SMX on day 24, completing a 42 day course (24 days intravenous and 18 days oral). Concurrently, three surgical interventions, including bowel resection, burr hole drainage, and Ommaya reservoir placement, were performed to address intestinal perforation and intracranial complications. At 4 months of corrected age, the infant showed age appropriate neurodevelopment with no recurrence or sequelae. This successful outcome underscores that prompt diagnostics, susceptibility driven antibiotic optimization, and a coordinated multidisciplinary surgical medical approach are essential for managing life threatening neonatal *Elizabethkingia anophelis* infections, even in the setting of severe multisystem involvement.

## 1 Introduction

*Elizabethkingia anophelis,* a Gram-negative, non-fermentative, aerobic bacillus belonging to the genus Elizabethkingia, represents a notable potential source of nosocomial infection. First isolated in 2011 from the midgut of Anopheles gambiae mosquitoes and initially reported in humans in 2013 (1, 2), this non-spore-forming, non-motile, oxidase-and indole-positive bacterium has been associated predominantly with sporadic infections, particularly rare in neonates. In this vulnerable population, infections typically occur in preterm infants with established risk factors, such as prior antibiotic exposure and prolonged hospitalization, and often manifest as nosocomial acquisitions (3, 4). Neonatal *Elizabethkingia anophelis* infection commonly presents as sepsis and meningitis, carrying high mortality rates and necessitating extended treatment. Survivors are at risk of significant sequelae, including hydrocephalus, hearing impairment, epilepsy, and developmental delay, which collectively impair long-term quality of life.

Elizabethkingia anophelis has been increasingly recognized as an opportunistic pathogen causing life-threatening infections in neonates, immunocompromised patients, and the elderly, accounting for 59.3%–96.2% of clinical Elizabethkingia genus isolates, though it was frequently misidentified as E. meningoseptica in earlier reports (5, 6). A recent systematic review of 45 studies reported that *E. anophelis* carries the highest mortality rate (39.1%) among Elizabethkingia species, followed by *E. miricola* (33.3%) and *E. meningoseptica* (11.8%); notably, neonatal cases achieved a 100% recovery rate with appropriate and timely treatment, whereas adults, especially those with comorbidities, had significantly poorer outcomes (7). In neonates, the infection most commonly manifests as fulminant sepsis and meningitis, with complications including ventriculitis, hydrocephalus, septic shock, acute pulmonary edema, and congestive cardiac failure; survivors may develop long-term neurological sequelae such as developmental delay and hearing impairment (3, 6). The pathogenesis of *E. anophelis* is attributed to 89 identified virulence-related genes, with biofilm-associated genes (fleQ, fleR, flmH, ugd), a type IV secretion system effector, and hemoglobin utilization mechanisms being universally conserved, promoting colonization, host-cell invasion, and bloodstream dissemination (8). These intrinsic virulence factors, combined with innate resistance to most β-lactams (including meropenem, ceftazidime, and cefepime), pose substantial challenges for empirical therapy and underscore the critical importance of early pathogen identification and susceptibility-guided antimicrobial selection (5, 7).

This report described the successful multidisciplinary management of a neonate with community-acquired *Elizabethkingia anophelis* sepsis, which rapidly progressed to intestinal necrosis and ventricular empyema. The case highlights the severe, invasive nature of this infection and provides practical insights for diagnosing and managing similar complex presentations in clinical practice.

## 2 Case report

### 2.1 Case history

The patient was a 10-day-old boy, Gravida 1, Para 2, at 35 ^+^ ^3^ weeks of gestation, cesarean delivered, with Apgar score 9 after 1 min and 10 after 5 min of birth. Birth parameters included a weight of 2.6 kg (55th percentile), head circumference of 32 cm (60th percentile), and length of 46 cm (50th percentile), with no abnormalities reported in amniotic fluid, placenta, or umbilical cord. He was on mixed feeding with adequate intake. On the 7th day of life, he developed maroon-colored mucoid stools, occurring 2–4 times daily without identifiable trigger, which did not improve after a switch to hydrolyzed formula. On the day of admission, he presented with fever (peak 39℃), abdominal distension, vomiting, and feeding refusal, without convulsions or high-pitched crying.

### 2.2 Investigations and treatment

On admission, the infant presented with a temperature of 38.2 ℃, irritability, tachypnea (64 breaths/min), tachycardia (165 bpm). Physical examination revealed lethargy, mottled skin, a tense anterior fontanelle (3 × 4 cm), and marked abdominal distension with visible superficial veins, guarding, and reduced bowel sounds (1/min); no rales or cardiac murmurs were detected, and limb muscle tone was decreased. Initial laboratory investigations showed elevated neutrophil percentage and C-reactive protein (CRP), while cerebrospinal fluid (CSF) analysis demonstrated pleocytosis (predominantly mononuclear), hypoglycorrhachia, hypochlororrhachia, and elevated protein. Hepatic/renal function, electrolytes, cardiac enzymes, and comprehensive immunology panels were unremarkable. Based on the clinical manifestations and laboratory findings, neonatal sepsis and necrotizing enterocolitis were suspected as the primary diagnosis.

Peripheral blood (1–2 mL) and CSF samples (2–3 mL) were collected prior to antimicrobial therapy and inoculated into separate aerobic and facultative anaerobic blood culture bottles (BacT/ALERT® SA, bioMérieux, France), then incubated in the automated BacT/ALERT® 3D system. The blood culture bottle tested positive approximately 8.6 h after collection, and the CSF culture bottle also flagged positive around 18 h after collection. Direct Gram staining of the positive broth showed scattered Gram-negative rods, which were either slender or coccobacillary in morphology. The positive broth was subcultured onto Columbia blood agar, chocolate agar, and MacConkey agar plates, then incubated at 35℃ in a 5% CO₂ atmosphere. After approximately 18–24 h of incubation, visible grayish-white, smooth colonies with entire edges and non-hemolytic characteristics grew. A single pure colony was picked for identification via MALDI-TOF mass spectrometry (microflex LT, Bruker Daltonics, Germany), which yielded a score of ≥2.00, and Elizabethkingia anophelis was identified on the second day of hospitalization. The culture and identification procedures described above follow the routine laboratory workflow for processing neonatal blood and CSF specimens in our microbiology department. Antimicrobial susceptibility testing (AST) was performed using Gram-negative broth microdilution panels (Thermo Fisher Scientific, USA) with *Pseudomonas aeruginosa ATCC 27853* as the quality control strain; panels were incubated at 35℃ in ambient air for 16–20 h. As summarized in Table 1, the *Elizabethkingia anophelis* isolate exhibited resistance to most tested antibiotics, including meropenem, ceftazidime, cefepime, and cefotaxime, indicating a multidrug-resistant profile. Notably, the isolate was susceptible to ciprofloxacin and levofloxacin, as well as to trimethoprim-sulfamethoxazole (TMP-SMX), and demonstrated intermediate susceptibility to cefepime and piperacillin/tazobactam. The complete data was shown in Table 1. Concurrently, on hospital day 2, the patient's plasma and CSF samples were sent for metagenomic next-generation sequencing (mNGS). DNA was extracted directly from the admission plasma and CSF specimens, after which libraries were constructed and sequenced on the MGISEQ-2000 platform with single-end 50-bp reads. Raw sequencing data were processed with fastp to remove low-quality reads and adapter sequences. The remaining reads were aligned to the human reference genome (hg38) via BWA to filter out host-derived sequences, and the remaining unmapped microbial reads were then aligned to the NCBI reference genome database for taxonomic classification. The sequencing run passed quality control, with Q30 rates of 92.10% for the plasma sample and 91.72% for the CSF sample. Ultimately, 5,532 stringent reads specifically mapping to E. anophelis were detected in the plasma sample with a 4.36% genome coverage, and 7,859 such reads were detected in the CSF sample with an 84.29% genome coverage. Both results had a confidence level of 99%, further confirming that this pathogen is the causative agent of the infection.

**Table 1:** Antimicrobial susceptibility testing results of the *elizabethkingia anophelis* clinical isolate.

No.	Antimicrobial agents	MIC（*μ*g/mL)	Breakpoints（μg/mL）	AST
1	Amikacin	32	≤16, ≥64	R
2	Ceftazidime	≥32	≤86, ≥32	R
3	Tobramycin	≥16	≤4, ≥16	R
4	Cefepime	16	≤8, ≥32	I
5	Cefoperazone/Sulbactam	≤8	≤8, ≥64	R
6	Ceftriaxone	64	≤8, ≥64	R
7	Ciprofloxacin	0.5	≤1, ≥4	S
8	Gentamicin	32	≤4, ≥16	R
9	Imipenem	≥16	≤4, ≥16	R
10	Levofloxacin	≤0.5	≤2, ≥8	S
11	Meropenem	≥32	≤4, ≥16	R
12	Minocycline	≤4	≤4, ≥16	R
13	Piperacillin/Tazobactam	32/4	≤16/4, ≥128/4	I
14	TMP-SMX	≤2/38	≤2/38, ≥4/76	S
15	Cefotaxime	64	≤8, ≥64	R
16	Aztreonam	≥64	≤8, ≥32	R

Antimicrobial susceptibility testing was carried out using Gram-negative broth microdilution panels (Thermo Fisher Scientific, USA). Pseudomonas aeruginosa ATCC 27853 was used for quality control.

MIC, minimum inhibitory concentration; S, susceptible; I, intermediate; R, resistant.

Supportive measures, including oxygen supplementation, nil per os (NPO), gastric decompression, intravenous immunoglobulin, and parenteral nutrition, were instituted upon admission, along with empirical meropenem and vancomycin. Once pathogen identification and AST were available, therapy was de-escalated to cefoperazone-sulbactam plus levofloxacin. However, persistent fever, progressive abdominal distension, and confirmed intestinal obstruction on day 3 prompted emergency laparotomy, which revealed hemorrhagic ascites, necrotic ileum near the ileocecal valve, and appendiceal perforation, leading to bowel resection and ileostomy (Figure 1). On day 9, new onset seizures and cranial magnetic resonance imaging (MRI) demonstrated meningeal enhancement, ventriculomegaly, ventricular empyema, and subdural effusions (Figure 2). Given ongoing bacteremia, the antibiotic regimen was changed to levofloxacin combined with TMP-SMX, followed by ventricular irrigation and burr-hole drainage on day 10, with continuous drainage maintained for one week. Fever subsided by day 11. Step down oral TMP-SMX was started on day 24. After drain removal, clinical and radiological signs of intracranial hypertension with progressive ventriculomegaly recurred, necessitating implantation of a ventricular Ommaya reservoir on day 34. A comprehensive clinical timeline illustrating dynamic changes in CRP, leukocyte count, antibiotic regimens, and major interventions is provided in Figure 3.

**Figure 1:**
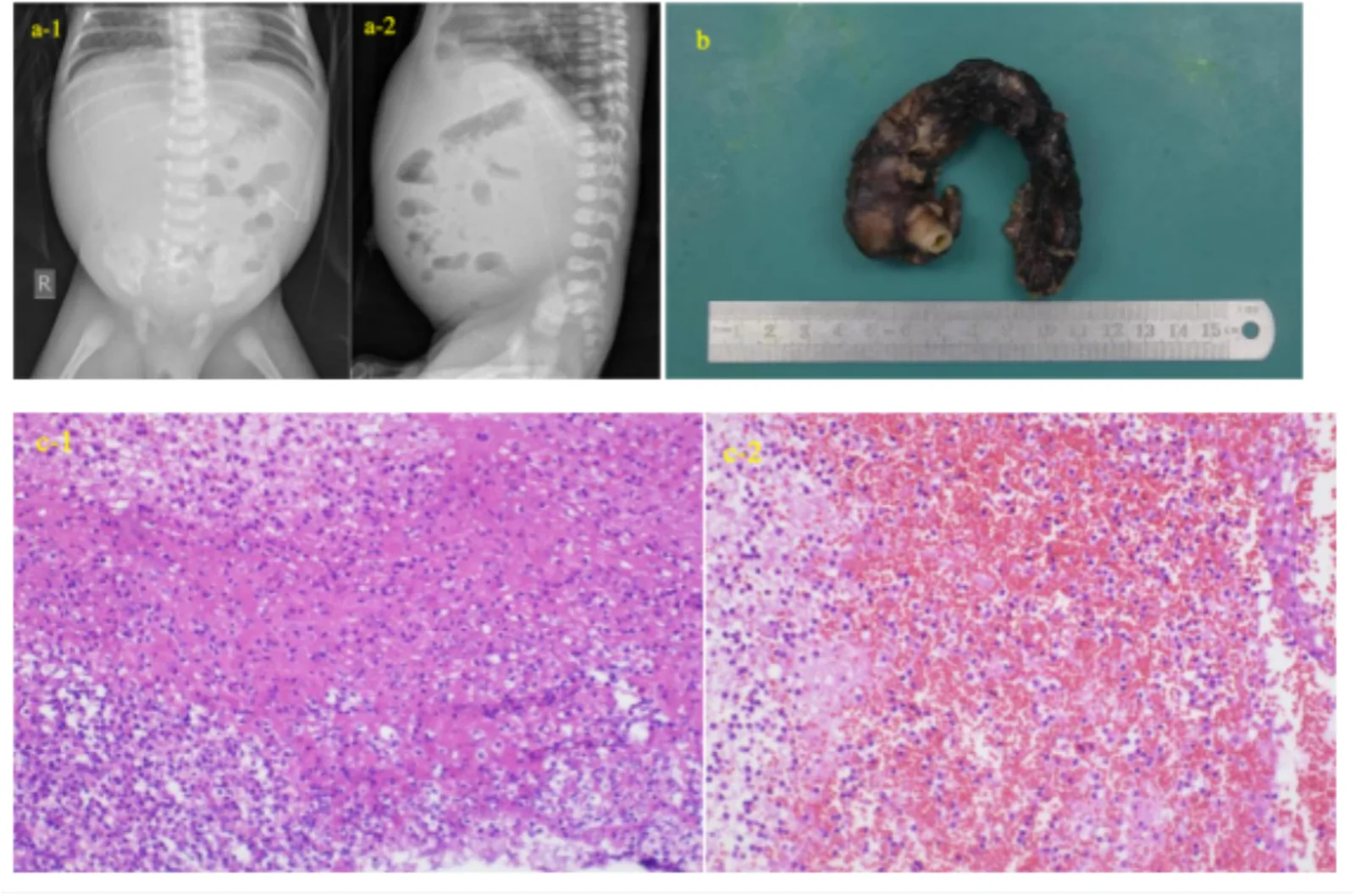
Abdominal radiography, surgical intestinal specimen, and histopathological findings. **al-a2**: Abdominal upright radiograph showing a paucity and uneven distribution of bowel gas. **b**: Surgical specimen showing a segment of necrotic colon. **c1-c2**: Photomicrographs of the resected intestine (HE staining × 200 magnification) demonstrating degeneration and sloughing of the intestinal mucosa, with focal areas of transmural hemorrhage and necrosis. There is extensive inflammatory infiltration predominantly composed of neutrophils, lymphocytes, and eosinophil.

**Figure 2:**
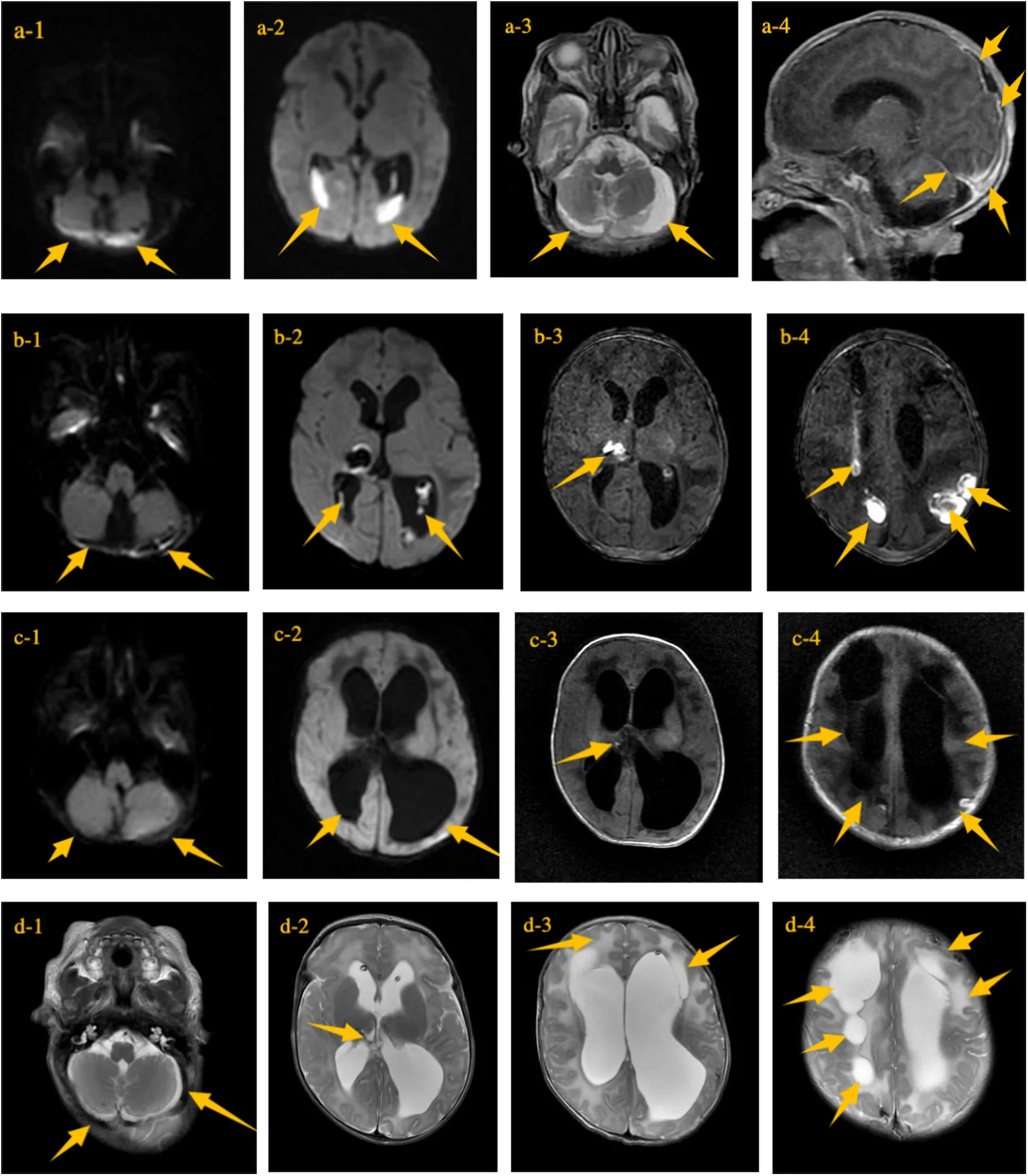
Figure 2 cranial magnetic resonance imaging (MRI) findings. **(a)** MRI in hospital day 9, Pre-burr hole drainage. al (DWI): empyema in the posterior fossa; a2(DWI): dilatation of the bilateral occipital horns of the lateral ventricles with intraventricular empyema; a3(T2WI): widening of the subarachnoid spaces surrounding the cerebellar hemispheres, consistent with subdural effusion; a4 (T1WI +C): thickened and enhanced leptomeninges in the posterior fossa, along the tentorium cerebelli, and over the parieto-occipital regions. **(b)** MRI in hospital day 15, post-burr hole drainage. b1 (DWI): resolution of the posterior fossa empyema compared with the previous al; b2 (DWI): dilatation of the bilateral occipital horns of the lateral ventricles with intraventricular empyema, partially resolved compared with a2; b3(T1WI): hemorrhagic foci in the right thalamus and within the choroid plexus of the lateral ventricles; b4(T1WI): multiple hemorrhagic foci in the bilateral cerebral parenchyma. **(c)** MRI in hospital day 32, pre-Ommaya reservoir placement. c1(DWI): the posterior fossa empyema has largely resolved compared with al and b1; c2(DWI): bilateral ventricles remain dilated, and the intraventricular empyema has largely resolved compared with a2 and b2; c3(T1WI): hemorrhagic foci in the right thalamus and choroid plexus have largely resolved compared with b3; c4(T1WI): multiple hemorrhagic foci in the bilateral cerebral parenchyma have mostly resolved compared with b4. **(d)** MRI in hospital day 41, post-Ommaya reservoir placement. d1(T2WI): slightly widened subarachnoid spaces around the cerebellar hemispheres, with subdural effusion largely resolved compared with al; d2 (T2WI): old hemorrhagic focus in the right thalamus with encephalomalacia and cystic changes; d3 (T2WI):bilateral ventriculomegaly with periventricular leukomalacia and cystic changes; d4 (T2WI): multiple foci of encephalomalacia and cystic changes in the bilateral cerebral parenchyma.

**Figure 3:**
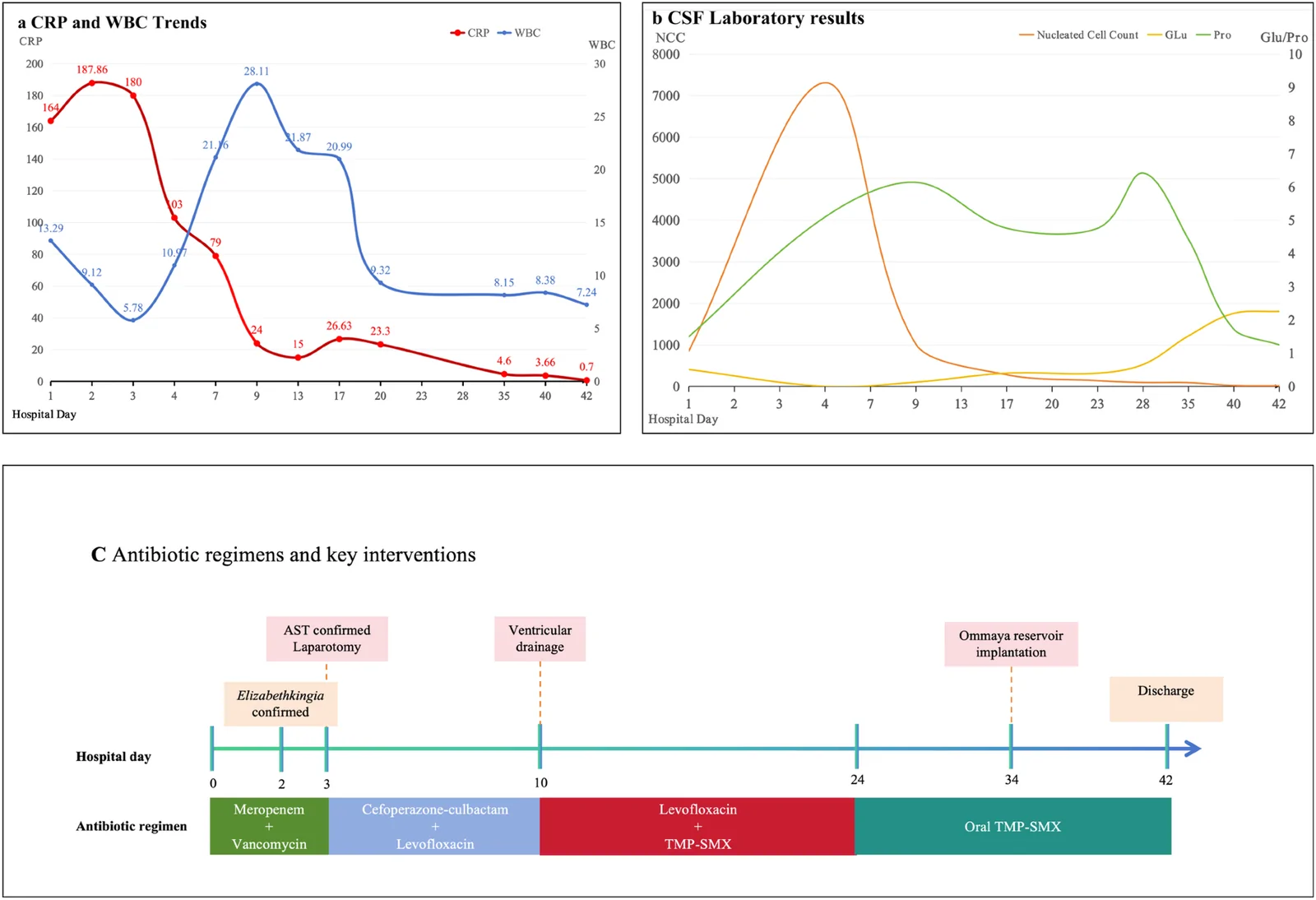
Clinical timeline. **(a)** Trends of CRP(mg/L), WBC(x10^9^/L); **(b)** cerebrospinal fluid laboratory results change; **(c)** antibiotic regimens and key interventions.

### 2.3 Outcome and follow-up

After clinical stabilization and normalization of cerebrospinal fluid parameters by day 40, the patient was discharged on day 42. Following discharge, the infant remained afebrile and seizure-free, with steady weight gain. Drainage was continued via the Ommaya reservoir, with serial imaging confirming stable ventriculomegaly and appropriate head-circumference progression. An ostomy closure was successfully performed 8 weeks after discharge. Multidisciplinary follow-up involving neurosurgery, neurology, rehabilitation, and child health services through 4 months of corrected age demonstrated age-appropriate physical and neurodevelopmental progress. However, long-term follow-up is necessary because this child may still show developmental problems (such as neurodevelopmental impairment and developmental delay) with age (3, 9).

## 3 Discussion

Delayed diagnosis of Elizabethkingia infections often leads to clinical deterioration (10, 11). In this case, both MALDI-TOF and mNGS identified the pathogen on the second day of hospitalization. MALDI-TOF provided the initial species-level identification, while mNGS independently confirmed this result and supplied additional genomic evidence, including genome coverage and specific read counts, which further supported the diagnosis of intracranial infection. Given the limited drug options available for neonates and the requirement for careful safety evaluation, the final treatment regimen had to be adjusted based on AST results. Although culture-based identification by MALDI-TOF was technically completed on day 2, this test follows our conventional microbiology workflow, where pathogen identification and AST results are typically compiled and released together as a single integrated report. For this reason, the formal report was not issued until the AST results became available on day 3. In contrast, the mNGS result was reported independently via a separate channel on day 2, providing the first pathogen-specific diagnostic information before the complete microbiology report was released. Accordingly, mNGS still plays a critical role in pathogen identification and clinical decision-making: it allowed the clinical team to formulate a clear treatment plan before receiving susceptibility results, enabling prompt adjustment once susceptibility testing confirmed drug sensitivity on day 3. Furthermore, early availability of pathogen information helps predict the risk of infection dissemination and the nature of potential complications, thereby facilitating timely surgical decision-making and source control. Therefore, the combination of MALDI-TOF and mNGS via parallel reporting pathways is a key prerequisite for successfully transitioning from empirical treatment to targeted combined surgical and medical treatment.

Following the selection of antibiotics based on AST results, the patient continued to exhibit persistent bacteremia and ventriculitis, reflecting a significant discrepancy between *in vitro* drug susceptibility and *in vivo* clinical efficacy. The proposed mechanism may involve the ability of *E. anophelis* to form biofilms (5), thereby enhancing antibiotic tolerance and leading to the ineffectiveness of conventionally selected drugs *in vivo*. Consequently, the treatment was adjusted to levofloxacin combined with TMP-SMX. The latter exhibits good cerebrospinal fluid penetration during meningeal inflammation. Notably, *in vitro* studies have demonstrated that levofloxacin combined with 1 × MIC of TMP-SMX can effectively delay or inhibit the selection of levofloxacin-resistant mutants in E. anophelis, supporting the rationale for this combination in treating persistent bacteremia (12). This adjustment proved clinically effective and successfully de-escalated to oral TMP-SMX therapy, demonstrating dynamic precision medicine based on pharmacokinetics and clinical response.

The infant presented with intestinal necrosis, ventriculitis, and hydrocephalus, all of which required surgical intervention, further confirming the importance of surgical debridement of deep infection foci in invasive infections. Intestinal necrosis was primarily attributed to gut hypoperfusion secondary to septic shock (13), and resection of the necrotic bowel segment combined with peritoneal lavage thoroughly eradicated the deep abdominal focus. For ventricular empyema, after burr-hole drainage to relieve acute intracranial hypertension, the temporary external drain was replaced with an Ommaya reservoir, which reduced the infection risk associated with long-term drainage and facilitated subsequent management of hydrocephalus and intracranial pressure regulation, thereby creating favorable conditions for neurological rehabilitation. Thus, the successful treatment of this case fundamentally benefited from the multidisciplinary collaborative model that ran throughout the entire treatment process.

## 4 Conclusion

This case demonstrates that for neonatal *Elizabethkingia anophelis* infection, early and precise diagnosis is the prerequisite for correct treatment, susceptibility-guided antimicrobial therapy forms the basis for infection control, and the timely recognition and surgical intervention for life-threatening complications such as intestinal necrosis and ventricular empyema are pivotal for reversing the clinical course. All these elements ultimately depend on efficient and collaborative integration by a multidisciplinary team for implementation. This study offers a valuable clinical reference for the comprehensive management of such complex and severe infections.

## Data Availability

The original contributions presented in the study are included in the article/Supplementary Material, further inquiries can be directed to the corresponding author.
